# Hybrid Chelator-Based PSMA Radiopharmaceuticals: Translational Approach

**DOI:** 10.3390/molecules26216332

**Published:** 2021-10-20

**Authors:** Hanane Lahnif, Tilmann Grus, Stefanie Pektor, Lukas Greifenstein, Mathias Schreckenberger, Frank Rösch

**Affiliations:** 1 Department of Chemistry—TRIGA Site, Johannes Gutenberg University Mainz, 55128 Mainz, Germany; halahnif@uni-mainz.de (H.L.); t.grus@uni-mainz.de (T.G.); greifenstein@curanosticum.de (L.G.); 2 Department of Nuclear Medicine, University Medical Center Mainz, 55131 Mainz, Germany; Stefanie.Pektor@unimedizin-mainz.de (S.P.); mathias.schreckenberger@unimedizin-mainz.de (M.S.); 3 Curanosticum Wiesbaden–Frankfurt, 65191 Wiesbaden, Germany

**Keywords:** prostate specific membrane antigen PSMA, hybrid chelator, radionuclide diagnosis and therapy

## Abstract

(1) Background: Prostate-specific membrane antigen (PSMA) has been extensively studied in the last decade. It became a promising biological target in the diagnosis and therapy of PSMA-expressing cancer diseases. Although there are several radiolabeled PSMA inhibitors available, the search for new compounds with improved pharmacokinetic properties and simplified synthesis is still ongoing. In this study, we developed PSMA ligands with two different hybrid chelators and a modified linker. Both compounds have displayed a promising pharmacokinetic profile. (2) Methods: DATA^5m^.SA.KuE and AAZTA^5^.SA.KuE were synthesized. DATA^5m^.SA.KuE was labeled with gallium-68 and radiochemical yields of various amounts of precursor at different temperatures were determined. Complex stability in phosphate-buffered saline (PBS) and human serum (HS) was examined at 37 °C. Binding affinity and internalization ratio were determined in *in vitro* assays using PSMA-positive LNCaP cells. Tumor accumulation and biodistribution were evaluated *in vivo* and *ex vivo* using an LNCaP Balb/c nude mouse model. All experiments were conducted with PSMA-11 as reference. (3) Results: DATA^5m^.SA.KuE was synthesized successfully. AAZTA^5^.SA.KuE was synthesized and labeled according to the literature. Radiolabeling of DATA^5m^.SA.KuE with gallium-68 was performed in ammonium acetate buffer (1 M, pH 5.5). High radiochemical yields (>98%) were obtained with 5 nmol at 70 °C, 15 nmol at 50 °C, and 60 nmol (50 µg) at room temperature. [^68^Ga]Ga-DATA^5m^.SA.KuE was stable in human serum as well as in PBS after 120 min. PSMA binding affinities of AAZTA^5^.SA.KuE and DATA^5m^.SA.KuE were in the nanomolar range. PSMA-specific internalization ratio was comparable to PSMA-11. *In vivo* and *ex vivo* studies of [^177^Lu]Lu-AAZTA^5^.SA.KuE, [^44^Sc]Sc-AAZTA^5^.SA.KuE and [^68^Ga]Ga-DATA^5m^.SA.KuE displayed specific accumulation in the tumor along with fast clearance and reduced off-target uptake. (4) Conclusions: Both KuE-conjugates showed promising properties especially *in vivo* allowing for translational theranostic use.

## 1. Introduction

Prostate-specific membrane antigen (PSMA) has become a very popular target in the diagnosis and treatment of prostate cancer in the last decade. PSMA is a glycoprotein with several functions originating from its glutamate-carboxypeptidase activity. In the central nervous system, PSMA acts as NAALADase, which cleaves the glutamate moiety from the neurotransmitter N-acetyl aspartyl glutamate. However, in the proximal small intestine, this enzyme, called folate hydrolase FOLH1, releases glutamate residues from poly-glutamated folate [1,2]. Besides these physiological functions, PSMA seems to play an important role in prostate carcinogenesis since it is highly expressed in prostate tumor cells. This expression correlates with the aggressiveness and invasiveness of the tumor [3,4,5], and is a major reason for choosing PSMA as a molecular target in the management of prostate cancer (PC).

Prostate cancer is the second most common cancer among men and the fifth leading cause of death worldwide [6,7]. However, early detection of PC in a localized stage can significantly reduce its mortality, leading to a 5-year survival rate of more than 90% [8]. In contrast, late-stage tumors are aggressive and almost resistant to available therapies. Metastatic castration-resistant prostate cancer (mCRPC) is one of the most aggressive forms of prostate cancer, with poor outcomes and restricted therapy options [9]. One of the most promising approaches herein is PSMA-targeted radioligand diagnosis and therapy. The unique characteristics of PSMA as a molecular target in combination with the small-molecule PSMA inhibitors as target vectors paved the way for the development of highly sensitive radiopharmaceuticals like the PET radioligand [^68^Ga]Ga-PSMA-11 and its therapeutic counterpart [^177^Lu]Lu-PSMA-617 [10,11].

One of the challenges in designing appropriate PSMA inhibitors for theranostic use is balancing the reduction of off-target accumulation in order to minimize the exposure and irradiation of excretory organs and other tissues where physiological PSMA expression is known, such as the salivary glands and the kidneys [12,13,14,15], with the development of PSMA ligands which can be easily synthesized and effectively labeled. To address some of these concerns, we developed AAZTA^5^.SA.KuE and DATA^5m^.SA.KuE.

Like all PSMA ligands, the herein described PSMA radiopharmaceuticals consist of three parts: chelator, linker moiety, and a KuE-based PSMA-targeting vector.

**Figure 1:**
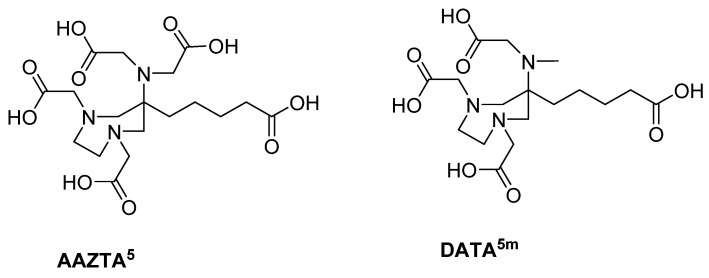
Bifunctional derivatives of the hybrid chelators AAZTA^5^ and DATA^5m^.

The chelator is responsible for the introduction of the radionuclide. In this study, the bifunctional hybrid chelators DATA^5m^ (6-pentanoic acid-6-aminoperhydro-1,4-diazapine-triacetate) and AAZTA^5^ (6-pentanoic acid-6-aminoperhydro-1,4-diazepine tetra-acetic acid) are used (Figure 1). With regard to radiometals, hybrid chelators combine the positive complexation properties of acyclic chelators, such as fast complexation kinetics at mild temperatures, with the advantages of cyclic chelators, such as prolonged complex stability [16,17]. In these structures, the two tertiary diazepine amines provide the cyclic component for complexation. Another amine outside the perhydro-1,4-diazepine backbone provides another complexation unit (acyclic component). The remaining complexation sites are provided by carboxy groups alkylated to the amines [16,18,19,20].

AAZTA^5^ shows ideal labeling properties for transition metals, such as scandium as well as for lanthanides, e.g., gadolinium and lutetium. Thus, AAZTA^5^ is suitable as a chelator for diagnostic use (e.g., scandium-44), as well as for therapeutic applications (e.g., lutetium-177) [17,21,22].

The DATA^5m^ chelator has optimal labeling properties at mild conditions for the generator-based PET nuclide gallium-68. Furthermore, the AAZTA and DATA chelators are also suitable for instant kit-labeling applications with e.g., lutetium-177 and gallium-68 [17,23].

Lysine-urea-glutamate (KuE) has been established as a PSMA inhibitor. KuE consists of lysine and glutamate which are both linked to each other via a urea unit. KuE is based on the natural PSMA-substrate NAAG, but cannot be cleaved by the enzyme [10]. Both PSMA-617 and PSMA-11 carry this structural unit as the PSMA-binding entity [24].

**Figure 2:**
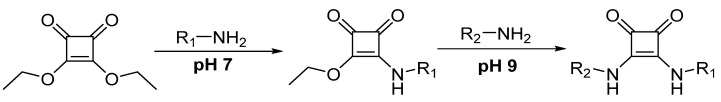
Asymmetric amidation of SADE with different amines. The reactions are driven by change in aromaticity of the different intermediates.

The third structural element found in PSMA radiopharmaceuticals is the linker moiety, connecting the chelator to the urea-based target vector. In addition to the function of coupling, these linkers are usually designed to improve the pharmacokinetics of the compounds [10]. These moieties can interact with the aromatic-binding region of the PSMA binding pocket, leading to an increase in the affinity of the PSMA ligand [25]. The coupling of KuE is achieved via the side-chain amine of the lysine. Usually, amide coupling reactions are used for this purpose. Alternatively, conjugation can be achieved by using square acid diethyl esters (SADE). This group allows two amines to be selectively coupled via asymmetric amidation, forming a squaramide. This simplifies the synthesis in so far as, for example, no protective group chemistry is required, as is the case with standard amide couplings. The coupling reaction is selective with amines only and by controlling the amidation of both squaric acid esters via pH [17,26,27,28,29]. The control of the asymmetric amidation via the pH value can be explained by the different aromaticity and thus the different mesomeric stabilities of the individual intermediates at the different pH values (Figure 2) [30,31,32].

With regard to PSMA radiopharmaceuticals, the use of squaric acid shows another advantage. Squaric acid has an aromatic character and can therefore interact with the aromatic binding region in the PSMA binding pocket resulting in an increased affinity. Greifenstein et al. recently demonstrated that a square amide containing DOTAGA-KuE derivative is comparable to the standard compounds PSMA-617 and PSMA-11 in terms of *in vitro* binding affinity, tumor accumulation, and *in vivo* kinetics [28].

## 2. Results

### 2.1. Organic Synthesis

#### 2.1.1. Synthesis of AAZTA^5^.SA.KuE Was according to Literature

**Figure 3:**
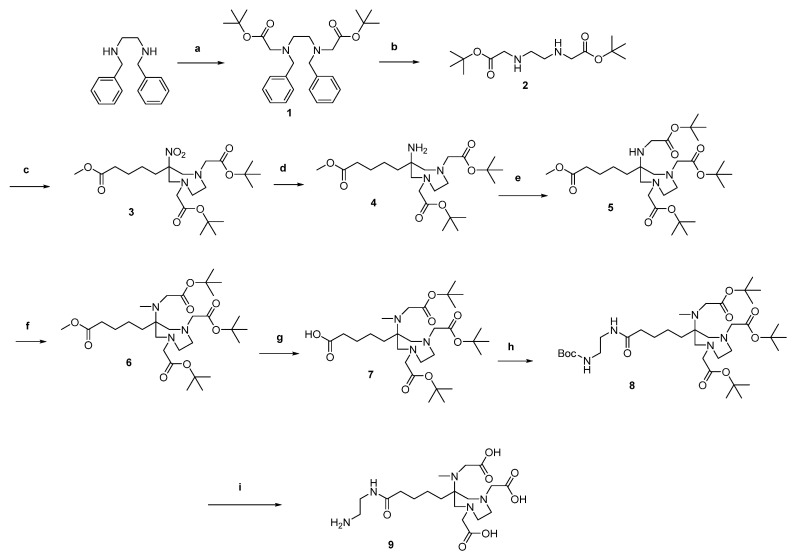
Synthesis of DATA^5m^: (a) tert-butyl bromoacetate, Na_2_CO_3_, MeCN, 96%; (b) Pd/C, EtOH, formic acid, H_2_, 98%; (c) paraformaldehyde, 2-nitrocyclohexanone, MeOH, 77%; (d) Raney^®^-Nickel, EtOH, H_2_, 72%; (e) *tert*-butyl bromoacetate, DIPEA, MeCN, 62%; (f) Formalin (37%), AcOH, NaBH_4_, ACN, 74%; (g) LiOH, dioxane/H_2_O, 84%; (h) tertbutyl(2aminoethyl)carbamate, HATU, DIPEA, ACN, 94%; (i) TFA/DCM, 1:1.

The synthesis of the DATA chelator is based on the synthesis described by Farkas et al. [16] and Greifenstein et al. [17]. It was synthesized according to Figure 3.

*N*,*N*′-dibenzylethyldiamines were first reacted with *tert*-butyl bromoacetate to give the di-alkylated compound **1**. The benzyl protecting groups were then removed by reduction. The diazepane **3** was formed by a double Mannich reaction. For this purpose, 2-nitrocyclohexanone was used, the ring of which was opened using the anion exchanger Amberlyst^®^ A21. In the following Mannich reaction, this ring-opened intermediate reacted with **2** to form the desired diazepane **3**.

After reduction of the nitro group (**4**), *tert*-butyl bromoacetate was added in an undercurrent to give the mono-alkylated compound **5**. The secondary amine of **5** was then methylated in a reductive amination. This led to the protected chelator DATA^5m^ **6**. In order to functionalize **6** with the target vector, however, it was necessary to introduce an ethylenediamine bridge. For this purpose, the methyl ester of **6** was saponified using lithium hydroxide (compound **7**) and the mono Boc-protected ethylenediamine was linked via an amide coupling to get **8**. After an acidic deprotection compound **9** could be conjugated to the target vector using squaric acid.

**Figure 4:**
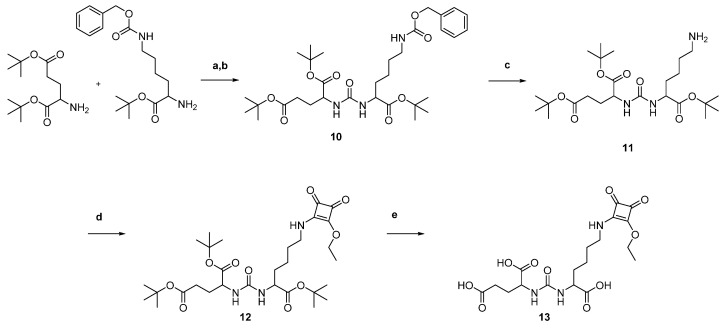
Synthesis of the PSMA inhibitor lysine-urea-glutamate-squaric acid monoester: (a) N(ε)-benzoyloxycarbonyl-l-lysine, triphosgene, triethylamine DCM, 0 °C; (b) l-glutamic acid di-*tert*-butyl ester hydrochloride, triethylamine, DCM, 41%; (c) Pd/C, MeOH, H_2_, 96%; (d) 3,4-dibutoxycyclobut-3-en-1,2-dione, 0.5 M phosphate buffer pH 7, ethyl acetate, 77%; (e) TFA/DCM, 1:1, 83%.

The PSMA inhibitor lysine-urea-glutamate (KuE) was synthesized and coupled to 3,4-dibutoxycyclobut-3-en-1,2-dione (SADE) according to Figure 4.

For the introduction of the urea unit, the amino group of the protected lysine was transformed into an isocyanate using triphosgene. The isocyanate was then reacted with *tert*-butyl protected glutamate and the protected PSMA inhibitor lysine-urea-glutamate **10** was obtained and followed by reductive deportation of the lysine side chain, yielding **11**. This compound was then coupled to SADE in phosphate buffer at pH 7. Acidic deprotection of the protected compound **12** led to the couplable PSMA inhibitor lysine-urea-glutamate-squaric acid monoester **13** (KuE.SAME).

**Figure 5:**
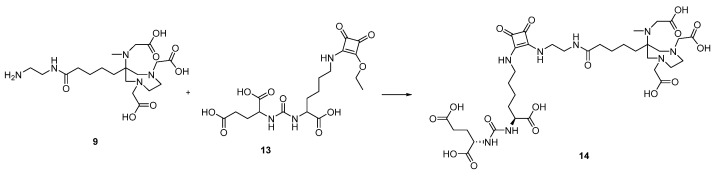
Synthesis of DATA^5m^.SA.KuE (**14**) 0.5 M phosphate buffer pH 9, 10%.

The free primary amine of DATA^5m^ (**9**) was then coupled to the free coupling side of KuE.SAME (**13**) in 0.5 M phosphate buffer at pH 9 to obtain the final compound DATA^5m^.SA.KuE (**14**) (Figure 5).

#### 2.1.2. Radiolabeling

Radiolabeling of AAZTA^5^.SA.KuE with scandium-44 and lutetium-177 was performed according to the literature [17].

**Figure 6:**
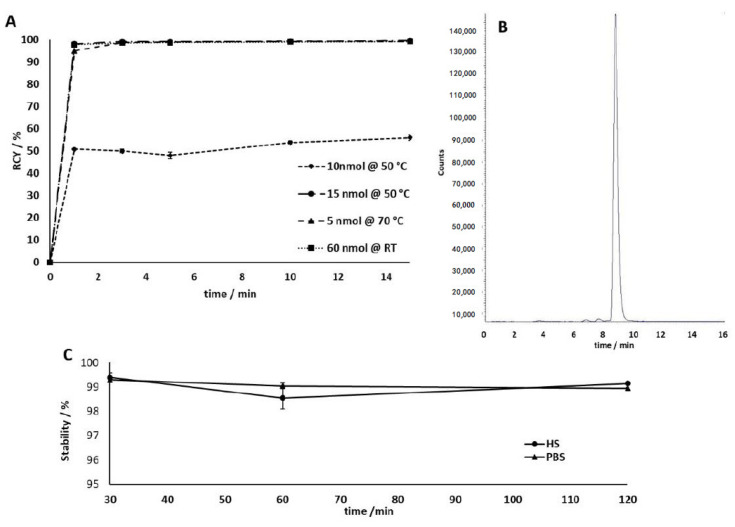
(**A**) Kinetic studies of ^68^Ga-radiolabeling of DATA^5m^.SA.KuE for various amounts of precursor and different temperatures. Labeling of 15 nmol at 50 °C, 5 nmol at 70 °C and 60 nmol at RT resulting in quantitative RCYs after one minute. Radiolabeling of 10 nmol at 50 °C results in a RCY of 56% after 15 minutes. (**B)** Radio-HPLC of [^68^Ga]Ga-DATA^5m^.SA.KuE. t_R_ (free gallium-68) = 2.0 min; t_R_ ([^68^Ga]Ga-DATA^5m^.SA.KuE) = 8.8 min. Radio-HPLC confirmed purity and high RCY of [^68^Ga]Ga-DATA^5m^.SA.KuE. (**C**) Stability studies for [^68^Ga] Ga-DATA^5m^.SA.KuE complex in human serum (HS) and phosphate buffered saline (PBS) of intact conjugate at different time points.

DATA^5m^.SA.KuE was radiolabeled with gallium-68 in ammonium acetate buffer (1 M, pH 5.5), varying amounts of precursor (5 nmol to 60 nmol), and different temperatures (room temperature to 70 °C). Labeling was carried out in triplicate with 30–50 MBq of gallium-68. Figure 6A shows the kinetic studies of the gallium-68-radiolabeling of DATA^5m^.SA.KuE. The lower the quantity of precursor used, the higher the temperature required to obtain quantitative radiochemical yields (RCY). Labeling of 10 nmol at 50 °C only achieved a RCY of 56% after 15 minutes. The increase to 15 nmol at 50 °C results in quantitative RCY (>99%). The increase of temperature even allowed the quantitative labeling (>99% RCY) of 5 nmol. Furthermore, 50 µg (60 nmol) can be radiolabeled in yields of over 99% with gallium-68 even at room temperature. The high radiochemical yield and high purity of [^68^Ga]Ga-DATA^5m^.SA.KuE was confirmed by radio-HPLC (Figure 6B).

Studies of the complex stability were performed in human serum (HS) and phosphate buffered saline (PBS). In both media, [^68^Ga]Ga-DATA^5m^.SA.KuE showed a stability of >98% over a period of 120 minutes (Figure 6C).

### 2.2. In Vitro Studies

#### 2.2.1. PSMA Binding Affinity

**Figure 7:**
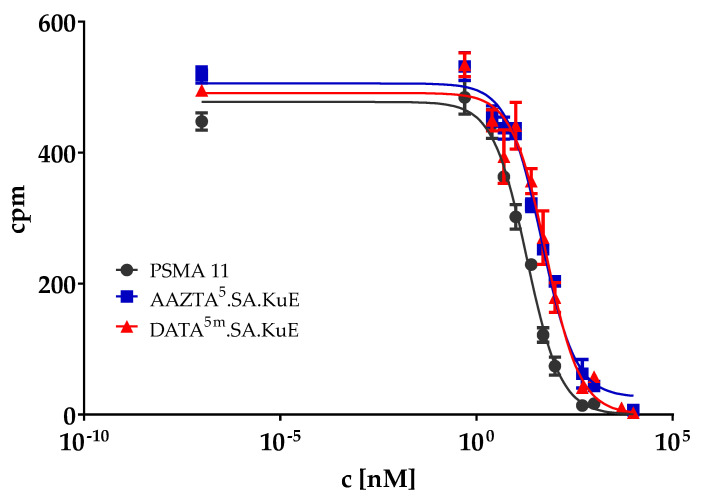
Inhibition curve of AAZTA^5^.SA.KuE, DATA^5m^.SA.KuE and PSMA-11. cpm: counts per minute.

The PSMA binding affinity of DATA^5m^.SA.KuE and AAZTA^5^.SA.KuE, as well as PSMA-11, was determined in a competitive radioligand assay using PSMA-positive LNCaP cells that were incubated with 0.75 nM [^68^Ga]Ga-PSMA-10 in the presence of 12 increasing concentrations of the non-labeled SA-conjugated compounds. The measured radioactivity values were plotted against the concentrations of the SA conjugates (Figure 7). IC_50_ values were determined using GraphPad Prism 9 (Table 1). AAZTA^5^.SA.KuE and DATA^5m^.SA.KuE showed similar binding affinities while PSMA-11 seems to have two-fold higher affinity *in vitro*.

#### 2.2.2. Internalization Ratio

**Figure 8:**
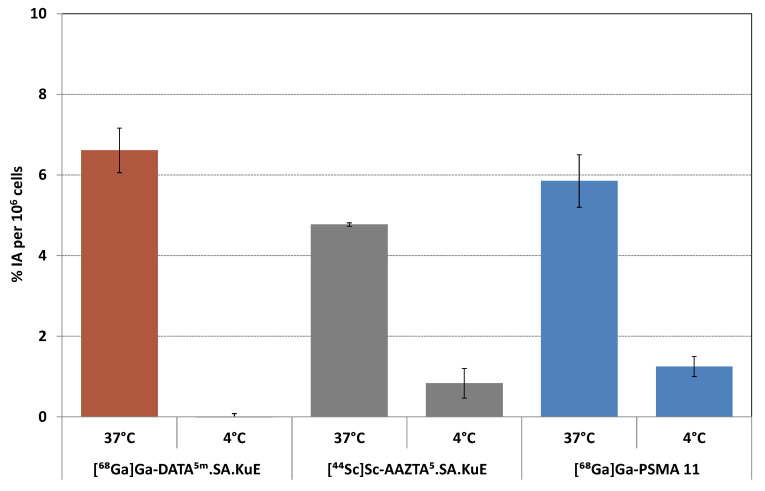
Internalization ratio of [^68^Ga]Ga-DATA^5m^.SA.KuE and [^44^Sc]Sc-AAZTA^5^.SA.KuE with [^68^Ga]Ga-PSMA-11 as reference; % injected dose per 10^6^ LNCaP cells.

PSMA ligands are internalized upon binding to PSMA, probably via clathrin-mediated endocytosis [33,34]. To determine the PSMA-specific cellular uptake of the developed PSMA ligands, we measured both the surface-bound and internalized radioactivity in PSMA-positive LNCaP cells at four different conditions; at 37 °C with and without blocking with the potent PSMA inhibitor PMPA (2-Phosphonomethyl pentanedioic acid) [35,36,37], and at 4 °C with and without blocking with PMPA. Results are plotted in Figure 8. [^68^Ga]Ga-DATA^5m^.SA.KuE displayed the highest internalization ratio 6.6 ± 0.6%, whereas the uptake fractions of [^44^Sc]Sc-AAZTA^5^.SA.KuE and [^68^Ga]Ga-PSMA-11 were slightly lower (4.8% and 5.2%, respectively). PSMA-specific uptake was mainly reduced at 4 °C.

### 2.3. Animal Studies

**Figure 9:**
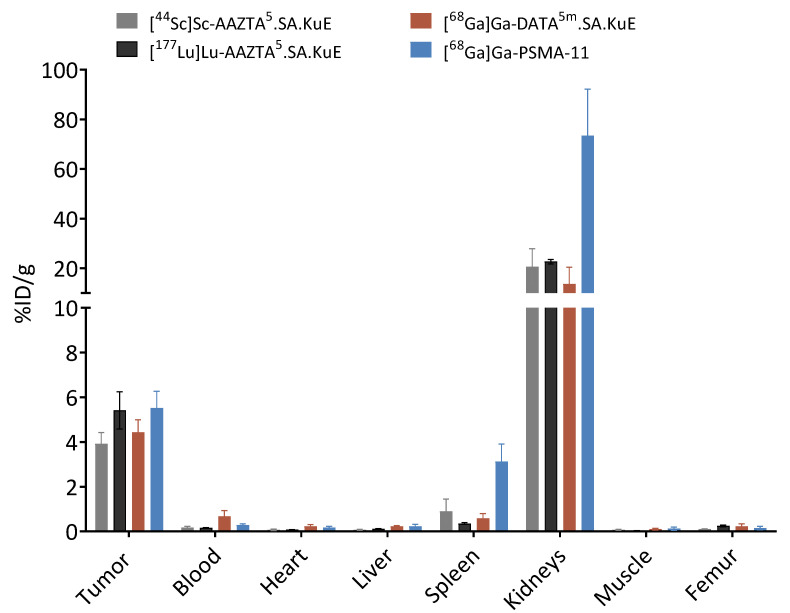
*Ex vivo* biodistribution data of [^44^Sc]Sc-AAZTA^5^.SA.KuE, [^177^Lu]Lu-AAZTA^5^.SA.KuE, [^68^Ga]Ga-DATA^5m^.SA.KuE and [^68^Ga]Ga-PSMA-11 in LNCaP tumor-bearing Balb/c nude mice 1 h p.i. %ID/g: % injected dose per gram. Values are mean ± SD.

**Figure 10:**
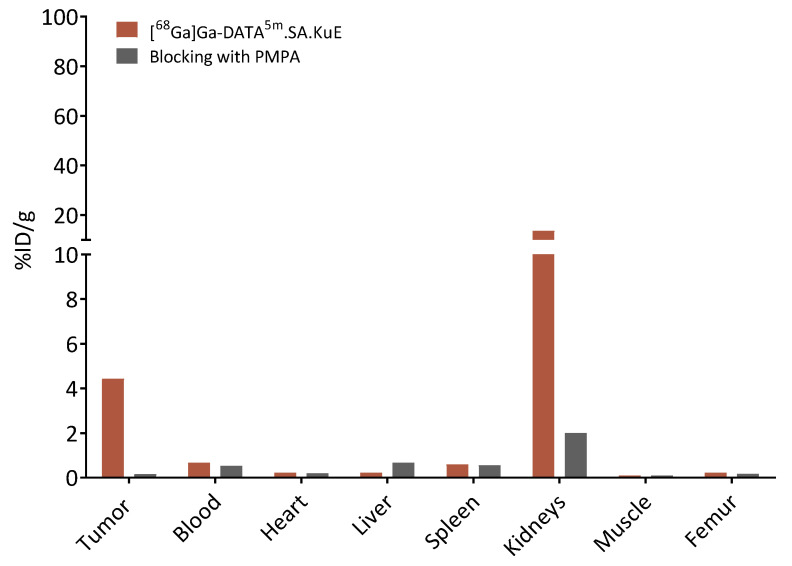
*Ex vivo* biodistribution of [^68^Ga]Ga-DATA^5m^.SA.KuE compared to organ accumulation after co-injection with an access PMPA.

In order to evaluate the *in vivo* behavior of the SA.KuE conjugates, an LNCaP-xenograft model was used. Labeling of AAZTA^5^.SA.KuE with the different nuclides scandium-44 and lutetium-177 seemed to have no impact on the pharmacokinetic properties of the conjugates, since there were no significant differences observed in the biodistribution data (Figure 9). Tumor accumulation values of all four compounds were similar, 3.92 ± 0.50% ID/g, 5.41 ± 0.83% ID/g, 4.43 ± 0.56% ID/g and 5.52 ± 0.75% ID/g for [^44^Sc]Sc-AAZTA^5^.SA.KuE, [^177^Lu]Lu-AAZTA^5^.SA.KuE, [^68^Ga]Ga-DATA^5m^.SA.KuE and [^68^Ga]Ga-PSMA-11 respectively. The higher kidney uptake of [^68^Ga]Ga-PSMA-11 (73.39 ± 18.77% ID/g) is noteworthy compared to the uptake of the SA.KuE conjugates (20.69 ± 7.24% ID/g, 22.70 ± 0.90% ID/g, 13.63 ± 6.81% ID/g for [^44^Sc]Sc-AAZTA^5m^.SA.KuE, [^177^Lu]Lu-AAZTA^5m^.SA.KuE and [^68^Ga]Ga-DATA^5^.SA.KuE respectively). Both tumor and kidney uptake of [^68^Ga]Ga-DATA^5^.SA.KuE were found to be PSMA-specific, since they could be blocked by co-injection of PMPA as seen in Figure 10.

**Figure 11:**
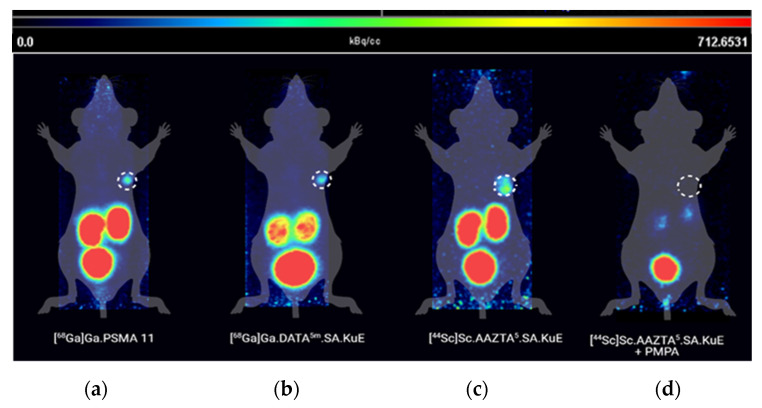
Maximum intensity projections of µPET scans 1 h p.i. of (**a**) [^68^Ga]Ga-PSMA-11, (**b**) [^68^Ga]Ga-DATA^5m^.SA.KuE, (**c**) [^44^Sc]Sc-AAZTA^5^.SA.KuE and (**d**) co-injection of [^44^Sc]Sc-AZTA^5^.SA.KuE and PMPA (tumor circled).

**Figure 12:**
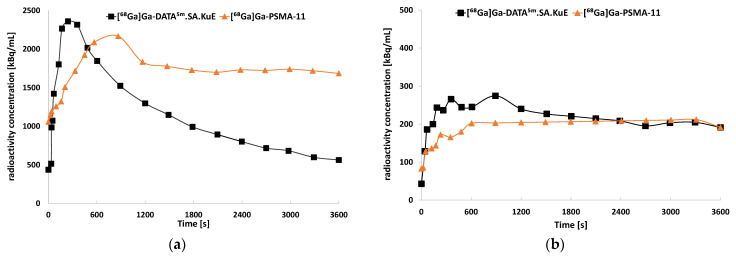
Time–activity curves of the uptake of [^68^Ga]Ga-DATA^5m^.SA.KuE and [^68^Ga]Ga-PSMA-11 in the kidneys (**a**) and the tumor (**b**) over the total period of the µPET scan.

To further understand the pharmacokinetics of the developed PSMA ligands, we performed µPET-scans with the same xenograft model (Figure 11). Tumor accumulation of all three compounds was very similar. The kidney uptake of [^68^Ga]Ga-DATA^5^.SA.KuE was remarkably lower than the reference compound [^68^Ga]Ga-PSMA-11. This finding correlates with the results obtained from the time–activity curves of both compounds (Figure 12). Herein, the radioactivity concentration of [^68^Ga]Ga-DATA^5m^.SA.KuE decreased continuously 10 min p.i. while the concentration in the tumor remained constant. However, the radioactivity concentration of [^68^Ga]Ga-PSMA-11 remained at a higher level during the period of the scan. As demonstrated in the µPET scans, uptake in the tumor as well as in the kidney was PSMA-specific. After co-injection of PMPA, almost no radioactivity could be detected (Figure 11).

## 3. Discussion

The discovery of PSMA as molecular target in the diagnosis and therapy of prostate cancer, as well as the application of radiolabeled PSMA inhibitors, have revolutionized the management of this disease resulting in a significant improvement especially in staging and assessment of prostate cancer [38]. Although several PSMA ligands have been developed over the last decades, the search for novel tracers with optimized pharmacokinetic properties particularly for therapeutic purposes is still present, since some of the clinically used PSMA radioligand therapeutics e.g., [^225^Ac]Ac-PSMA-617 display some severe side effects, like xerostomia [12,13,39].

To determine the effect of the chelator on the PSMA binding affinity and the internalization ratio of PSMA ligands, we synthesized two PSMA inhibitors with different hybrid chelators. In the cell-based assays, both DATA^5m^.SA.KuE and AAZTA^5^.SA.KuE showed similar binding affinity and internalization ratios, indicating that an exchange of DATA^5m^ against AAZTA^5^ had no impact on either the binding affinity or on the internalization ratio in PSMA-positive LNCaP cells. These findings correlate with the results published by Sinnes et al., who investigated the influence of the exchange of DOTA chelator in DOTA-PSMA-617 against AAZTA^5^. Both DOTA-PSMA-617 and AAZTA^5.^-PSMA-617 displayed similar *in vitro* binding affinities and internalization ratios in LNCaP cells [40]. However, the reported binding affinities and internalization ratios of AATA^5^-PSMA-617 and DOTA-PSMA-617 were higher than those of the SA.KuE conjugates. In particular, [^44^Sc]Sc-PSMA-617 seems to display high PSMA-binding affinity as published by several groups [41,42]. Since PSMA-617 was not commercially available at the time this study was performed, PSMA-11 was used as reference.

However, PSMA-11 displayed also higher binding affinity *in vitro* which could be due to the better interaction with the PSMA binding pocket. In contrast, the internalization ratio of PSMA-11 was similar to these of the SA.KuE-conjugates. Interestingly, the investigated internalization fraction of [^68^Ga]Ga-PSMA-11 was noticeably lower compared to the ratio described in literature [42,43] which could be due to differences in study design and setup. The PSMA-specificity of binding and uptake in LNCaP cells and LNCaP tumors could be demonstrated for all herein investigated PSMA-inhibitors by blocking PSMA receptors with the potent inhibitor PMPA.

In order to evaluate the pharmacokinetic behavior of our compounds and to compare them with PSMA-11, we performed animal studies using an LNCaP xenograft model.

AAZTA^5^.SA.KuE was labeled with the positron emitter scandium-44 and β^−^-emitterlutetium-177. Both radiotracers displayed similar biodistribution data, indicating that both isotopes do not impact the pharmacokinetic properties of the PSMA radioligand. This result makes this pair ideal for theranostic use. In addition, [^68^Ga]Ga-DATA^5m^.SA.KuE equally showed a promising biodistribution profile and a good imaging contrast. Surprisingly, although PSMA-11 showed a two-fold higher binding affinity *in vitro*, tumor accumulation was similar to the SA.KuE-conjugates. Furthermore, the kidney uptake of [^68^Ga]Ga-PSMA-11 was significantly higher than the SA.KuE-conjugated compounds. [^68^Ga]Ga-DATA^5m^.SA.KuE, [^44^Sc]Sc-AAZTA^5^.SA.KuE and [^177^Lu]Lu-AAZTA^5^.SA.KuE. Thus, these compounds seem to display a rapid renal clearance along with a good tumor accumulation. However, the tumor uptake of the SA.KuE conjugates was lower than that of the gallium-68 and lutetium-177 labeled PSMA-617 radioligands [10,11]. Ghiani et al. recently described a novel scandium-44 labeled PSMA radioligand with even higher tumor accumulation than the PSMA-617 counterpart [41]. Nevertheless, a direct comparison between the presented results and those reported by other groups is not possible because of the differences in xenograft models and experimental setups.

## 4. Materials and Methods

### 4.1. General

All chemicals were purchased from Sigma-Aldrich (St. Louis, MO, USA), Merck (Kenilworth, NJ, USA), Fluka (Buchs, Switzerland), AlfaAesar (Haverhill, MA, USA), VWR (Radnor, PA, USA), AcrosOrganics (Geel, Belgium), TCI (Portland, OR, USA), Iris Biotech (Marktredwitz, Germany) and Fisher Scientific (Hampton, NH, USA) and used without purification. Dry solvents were obtained from Merck and VWR, deuterated solvents for NMR spectra from Deutero. Thin layer chromatography was performed with silica gel 60 F254 coated aluminum plates from Merck. Evaluation was carried out by fluorescence extinction at λ = 254 nm and staining with potassium permanganate. The radio TLCs were evaluated using a CR-35 Bio test imager (Elysia-Raytest, Angleur, Belgium) from Raytest and the AIDA software (Elysia-Raytest, Angleur, Belgium). The ^1^H and ^13^C NMR measurements were performed on an Avance III HD 300 spectrometer (Bruker Corporation, Billerica, MA, USA) (300 MHz, 5mm BBFO sample head with z-gradient and ATM and BACS 60 sample changer), an Avance II 400 (Bruker Corporation, Billerica, MA, USA) (400 MHz, 5 mm BBFO sample head with z-Gradient and ATM and SampleXPress 60 sample changer), and an Avance III 600 spectrometer (Bruker Corporation, Billerica, MA, USA) (600 MHz, 5mm TCI CryoProbe sample head with z-Gradient and ATM and SampleXPress Lite 16 sample changer). The LC/MS measurements were performed on an Agilent Technologies 1220 Infinity LC system coupled to an Agilent Technologies 6130B Single Quadrupole LC/MS system. Semi-preparative HPLC purification was performed on a 7000 series Hitachi LaChrom (Hitachi, Chiyoda, Japan).

### 4.2. Organic Synthesis

DATA^5m^ was synthesized according to the procedure described by Farkas et al. and Greifenstein et al. [17].

*N*,*N*′-Dibenzyl-*N*,*N*′-di-(tert-butylacetate)-ethylendiamine (**1**)

*N*,*N*′-dibenzylethylendiamine (2.90 mL, 3.00 g, 12.50 mmol) and sodium carbonate (5.10 g, 48.70 mmol) were dissolved in acetonitrile (50 mL) and stirred for 30 min at room temperature. *Tert*-butyl bromoacetate (3.60 mL, 4.60 g, 23.70 mmol) in acetonitrile (10 mL) was added dropwise at room temperature. The reaction mixture was stirred overnight at 90 °C and filtered. The solvent was evaporated under reduced pressure. The product was purified by column chromatography (hexane/ethyl acetate; 6:1, R_f_ = 0.37) and obtained as a colorless solid (5.73 g, 12.2 mmol, 96%).

^1^H-NMR (400 MHz, CDCl_3_): δ [ppm] = 7.34–7.21 (m, 10H), 3.78 (s, 4H), 3.26 (s, 4H), 2.82 (s, 4H), 1.44 (s, 18H).

^13^C-NMR (400 MHz, CDCl3): δ [ppm] = 171.03, 139.18, 129.05, 128.30, 127.10, 80.86, 58.39, 55.27, 51.73, 28.24.

MS (ESI^+^): 469.4 [M + H]^+^, calculated for C_28_H_40_N_2_O_4_: 468.30 [M]^+^.

*N*,*N*′-di-(*tert*-butylacetate)-ethylendiamine (**2**)

Product **1** (2.3 g; 5.60 mmol) was dissolved in abs ethanol (15 mL) and formic acid (0.43 mL, 0.52 g, 11.0 mmol). To this solution palladium on activated carbon (416 mg, 16% wt) was added and the solution was saturated, kept and stirred overnight with hydrogen. Pd/C was filtered over celite and the solvent was evaporated under reduced pressure. The product (1.58 mg, 5.5 mmol, 98%) was used without further purification.

MS (ESI^+^): 289.3 [M + H]^+^, calculated for C_14_H_28_N_2_O_4_: 288.36 [M]^+^.

1,4-Di(*tert*-butylacetate)-6-methyl-6-nitroperhydro-1,4-diazepane (**3**)

2-Nitrocyclohexanone (1.70 g, 12 mmol) and Amberlyst^®^ A21 (2 mass equivalents) were dissolved in methanol (30 ml) and stirred at 90 °C for 1 h. Paraformaldehyde (1.30 g, 42.3 mmol) and Product (2) (3.50 g, 12 mmol) were added. The solution was heated overnight under reflux. The solvent was evaporated under reduced pressure. The product was purified by column chromatography (hexane/ethyl acetate; 2:1, R_f_ = 0.33) and obtained as a yellowish oil (4.52 g, 9.28 mmol, 77%).

^1^H-NMR (400 MHz, CDCl_3_): δ [ppm] = 3.65 (s, 3H), 3.60 (d, *J* = 14,6 Hz, 2H), 3.45 (d, *J* = 17.3 Hz, 2H), 3.30 (d, *J* = 17.3 Hz, 2H), 3.12 (d, *J* = 14.6 Hz, 2H), 2.84 (m, 4H), 2.27 (t, 2H), 1.83 (m, 2H), 1.57 (m, 2H), 1.46 (s, 18H), 1.18 (m, 2H).

^13^C-NMR (400 MHz, CDCl_3_): δ [ppm] = 173.73, 170.92, 95.12, 81.31, 61.57, 61.18, 56.87, 51.68, 37.27, 33.71, 28.35, 24.82, 22.99.

MS (ESI^+^): 488.3 [M + H]^+^, calculated for C_23_H_41_N_3_O_8_: 487.29 [M]^+^.

1,4-Di(*tert*-butylacetate)-6-methylpentanoate-6-amino-perhydro-1,4-diazepane (**4**)

Compound **3** (4.50 g, 9.30 mmol) was dissolved in abs. ethanol (40 mL). Raney^®^ nickel was added and the solution was saturated, kept and stirred for four days with hydrogen at 40 °C. The nickel was filtered over celite and the solvent was evaporated under reduced pressure. Compound **4** (3.92 g, 8.60 mmol, 72%), was obtained as a greenish oil and used without further purification.

MS (ESI^+^): 458.3 [M + H]^+^, calculated for C_23_H_43_N_3_O_6_: 457.32 [M]^+^.

1,4-Di(*tert*-butylacetate)-6-methylpentonate-6-amino-*tert*-butylacetate-perhydro-1,4-diazepane (**5**)

Compound **4** (1.30 g, 2.84 mmol) and *N*,*N*-diisopropylethylamine (483 µL, 367 mg, 2.84 mmol) were dissolved in dry acetonitrile (20 mL) and stirred for 20 min at room temperature. *Tert*-butyl bromoacetate (538 µL, 720 mg, 3.69 mmol) was added dropwise and stirred overnight at room temperature. The solvent was evaporated under reduced pressure. The product was purified by column chromatography (cyclohexane/ethyl acetate; 3:1 + 3% trimethylamine, R_f_ = 0.34) and obtained as a yellowish oil (1.01 g, 1.77 mmol, 62%).

^1^H-NMR (400 MHz, CDCl_3_): δ [ppm] = 3.65 (s, 3H), 3.29 (s, 4H), 3.21 (s, 2H), 2.83–2.60 (m, 8H), 2.30 (dd, *J* = 8.9, 6.3 Hz, 2H), 1.90 (s br, 1H), 1.62–1.54 (m, 2H), 1.46 (s, 9H), 1.44 (s, 18H), 1.32–1.23 (m, 4H).

MS (ESI^+^): 572.4 [M + H]^+^, calculated for C_29_H_53_N_3_O_8_: 571.38 [M]^+^.

1,4-Di(*tert*-butylacetate)-6-methylpentonate-6-(amino(methyl)-*tert*-butylacetate)-perhydro-1,4 diazepane (**6**)

Compound **5** (1.00 g, 1.75 mmol), formalin solution (482 µL, 526 mg, 6.47 mmol), and acetic acid (300 µL, 315 mg, 5.25 mmol) were dissolved in dry acetonitrile (20 mL) and stirred at room temperature for 30 min. Sodium borhydride (200 mg, 5.29 mmol) was added portion-wise over 30 min. The reaction solution was stirred for 2 hours at room temperature. Water (25 mL) was added and extracted with chloroform (4 × 50 mL). The organic phase was separated and dried over sodium sulfate and evaporated under reduced pressure. The product was purified by column chromatography (cyclohexane/ethyl acetate; 5:1 + 2% trimethylamine, R_f_ = 0.28) and obtained as colorless oil (0.76 g, 1.39 mmol, 74%).

^1^H-NMR (400 MHz, CDCl_3_): δ [ppm] = 3.65 (s, 3H), 3.42 (s, 2H), 3.32–3.18 (m, 4H), 2.93 (d, *J* = 14.0 Hz, 2H), 2.83–2.73 (m, 2H), 2.70–2.58 (m, 4H), 2.35−2.24 (m, 5H), 1.63–1.48 (m, 4H), 1.45 (s, 9H), 1.44 (s, 18H), 1.41–1.22 (m, 2H).

MS (ESI^+^): 586.4 [M + H]^+^, calculated for C_30_H_55_N_3_O_8_: 585.40 [M]^+^.

1,4-Di(*tert*-butylacetate)-6-pentanoicacid-6-(amino(methyl)-*tert*-butylacetate)-perhydro-1,4-diazepane (**7**)

Compound **6** (0.75 g, 1.28 mmol) was dissolved in a 1,4-dioxane/water (2:1) mixture. Then, 1 M lithium hydroxide solution (1.92 mL, 1.92 mmol) was added and stirred for 7 days. After 2, 4, and 6 days 1 M lithium hydroxide solution (0.32 mL, 0.32 mmol) was added. 1,4-dioxane was evaporated under reduced pressure. The remaining water phase was extracted with chloroform (5 × 50 mL). The organic phase was washed with 1 M sodium hydrogen carbonate solution (25 mL) and brine (2 × 25 mL) and dried over sodium sulfate and evaporated under reduced pressure. The product (615 mg, 1.07 mmol, 84%) was obtained as a yellowish oil.

^1^H-NMR (400 MHz, CDCl_3_): δ [ppm] = 3.44 (s, 2H), 3.25 (d, *J* = 2.2 Hz, 4H), 2.93 (d, *J* = 14.0 Hz, 2H), 2.82–2.73 (m, 2H), 2.71–2.61 (m, 4H), 2.34 (t, *J* = 7.7 Hz, 2H), 2.27 (s, 3H), 1.65–1.51 (m, 4H), 1.45 (s, 18H), 1.44 (s, 9H), 1.43–1.21 (m, 2H).

^13^C-NMR (400 MHz, CDCl_3_): δ [ppm] = 178.46, 172.53, 170.98, 81.02, 80.44, 77.36, 62.86, 62.59, 62.48, 59.02, 54.21, 37.49, 36.92, 34.17, 28.37, 28.27, 25.71, 21.97.

MS (ESI^+^): 572.4 [M + H]^+^, calculated for C_29_H_53_N_3_O_8_: 571.38 [M]^+^.

1,4-Di(*tert*-butylacetate)-6-((5-(2-((2-ethoxy-3,4-dioxocyclobut-1-en-1yl)amino-ethyl)amino)-5-oxopentyl)-6-(amino(methyl)-tert-butylacetate)-perhydro-1,4-diazepane (**8**)

Compound **7** (100 mg, 0.175 mmol), HATU (66.5 mg, 0.175 mmol), and DIPEA (90 µl, 69 mg, 0.525 mmol) were dissolved in dry acetonitrile (2 mL) and stirred for 15 min at room temperature. *Tert*-butyl(2-aminoethyl) carbamate (45 µL, 46 mg, 0.280 mmol) was added to the solution and stirred over night at room temperature. The solvent was evaporated under reduced pressure. The product was purified by column chromatography (dichloromethane/methanol; 20:1, R_f_ = 0.22) and obtained as a colorless oil (118.4 mg, 0.166 mmol, 94%).

^1^H-NMR (400 MHz, CDCl_3_): δ [ppm] = 6.34 (br, 1H), 5.26 (br, 1H), 3.60 (s, 4H), 3.38–3.34 (m, 2H), 3.26-3.24 (m, 2H), 3.21 (s, 4H), 2.96 (d, *J* = 14,1 Hz, 2H), 2.75–2.63 (m, 2H), 2.66-2.63 (m, 2H), 2.59 (d, *J* = 14,1 Hz, 2H), 2.19 (t, 2H), 1.62-1.53 (m, 4H), 1.43 (s, 18H), 1.42 (s, 27H), 1.28-1.20 (m, 2H).

^13^C-NMR (400 MHz, CDCl_3_): δ [ppm] = 174.38, 173.31, 172.80, 165.88, 82.85, 82.77, 63.44, 62.48, 62.05, 55.48, 54.47, 47.11, 40.81, 39.87, 35.55, 29.82, 28.53, 28.32, 28.14, 27.91, 26,17, 23.41.

MS (ESI^+^): 714.5 [M + H]^+^, calculated for C_36_H_67_N_5_O_9_: 713.49 [M]^+^.

1,4-Di(acetate)-6-((5-(2-(aminoethyl)amino)-5-oxopentyl)-6-(amino(methyl)-acetate)-perhydro-1,4-diazepane (**9**)

Compound **8** (20 mg, 0.028 mmol) was dissolved in a solution of dichloromethane and triflouroacetic acid (2 mL, 1:1) and stirred over night at room temperature. The solvent was evaporated under reduced pressure and used without further purification.

MS (ESI^+^): 446.2 [M + H]^+^, calculated for C_19_H_35_N_5_O_7_: 445.25 [M]^+^.

2-[3-(5-benzyloxycarbonylamino-1-tert-butoxycarbonyl-pentyl)-ureido]-pentanedioic acid di-*tert*-butyl ester (**10**)

Triphosgene (420 mg, 1.40 mmol) was dissolved in dichloromethane (5 mL) and cooled to 0 °C. A solution of N(ε)-benzoyloxycarbonyl-l-lysine (1.42 g, 3.80 mmol) and triethylamine (1.05 mL, 765 mg, 7.60 mmol) in dichloromethane (25 mL) was added dropwise over a period of 3 hours at 0 °C. The reaction mixture was stirred for 40 minutes and l-glutamic acid di-*tert*-butyl ester hydrochloride (1.13 g, 3.80 mmol) and triethylamine (1.05 mL, 765 mg, 7.60 mmol) in dichloromethane (20 mL) was added. The solution was stirred over night at room temperature. The solution was evaporated under reduced pressure. Ethyl acetate (25 mL) was added. The organic layer was washed with saturated NaHCO_3_-solution (2 × 10 mL) and brine (2 × 10 ml), dried over sodium sulfate and evaporated under reduced pressure. The residue was purified by column chromatography (hexane/ethyl acetate; 20:1, R_f_ = 0.26) and the product was obtained as a colorless oil (357.6 mg, 0.58 mmol, 41%).

^1^H-NMR (300 MHz, CDCl_3_): δ [ppm] = 7.38–7.22 (m, 5H), 5.16 (d, *J* = 13.5 Hz, 1H), 5.09 (d, *J* = 3.2 Hz, 2H), 4.32 (dt, *J* = 7.5, 5.2 Hz, 2H), 3.16 (s, 2H), 2.40–2.15 (m, 2H), 1.93–1.68 (m, 2H), 1.43 (m, 29H).

^13^C-NMR (300 MHz, CDCl_3_): δ [ppm] = 172.54, 172.25, 172.15, 157.09, 156.61, 136.67, 128.47, 128.04, 128.01, 82.29, 81.84, 80.65, 77.24, 66.56, 53.38, 53.03, 40.63, 32.53, 31.52, 29.36, 28.28, 28.07, 28.00, 22.26.

MS (ESI^+^): 622.4 [M + H]^+^, 644.4 [M + Na]^+^, calculated for C_32_H_51_N_3_O_9_: 621.36 [M]^+^.

2-[3-(amino-1-tert-butoxycarbonyl-pentyl)-ureido]-pentanedioic acid di-*tert*-butyl ester (**11**)

Compound **10** (337.6 mg, 0.55 mmol) was dissolved in methanol (3 mL). To this, solution palladium on activated carbon (22 mg) was added and the solution was saturated, kept and stirred overnight with hydrogen. Pd/C was filtered over celite and the solvent was evaporated under reduced pressure. The product (260 mg, 0.53 mmol, 96%) was used without further purification.

^1^H-NMR (300 MHz, CDCl_3_): δ [ppm] = 5.48 (dd, *J* = 10.3, 8.1 Hz, 2H), 4.31 (dd, *J* = 5.7, 2.4 Hz, 2H), 2.77 (t, *J* = 6.6 Hz, 2H), 2.36–2.25 (m, 2H), 2.05 (ddd, *J* = 7.1, 5.9, 2.1 Hz, 1H), 1.92–1.68 (m, 2H), 1.44 (d, *J* = 7.1 Hz, 33H).

^13^C-NMR (300 MHz, CDCl_3_): δ [ppm] = 172.61, 172.47, 157.05, 82.07, 81.67, 80.55, 53.39, 52.99, 41.12, 32.40, 31.66, 31.43, 28.28, 28.08, 28.02, 22.20.

MS (ESI^+^): 488.3 [M + H]^+^, calculated for C_24_H_45_N_3_O_7_: 487.33 [M]^+^.

2-[3-(2-(2-ethoxy-3,4-dioxo-cyclobut-1-en-1yl)amino-1-*tert*-butoxycarbonyl-pentyl)-ureido]-pentanedioic acid di-tert-butyl ester (**12**)

Compound **11** (260 mg, 0.53 mmol) was dissolved in 0.5 M phosphate buffer (pH 7, 2 mL), 3,4-dibutoxycyclobut-3-en-1,2-dione (82 µL, 95 mg, 0.53 mmol) was added and the pH was adjusted to 7. Ethyl acetate (1 mL) was added and stirred overnight. The solvent was then removed via lyophilization and ethyl acetate (2 mL) was added. The solution was then filtered and the solvent was removed under reduced pressure. The product (248 mg, 0.41 mmol, 77%) was obtained as a colorless oil and used without further purification.

^1^H-NMR (300 MHz, CDCl_3_): δ [ppm] = 4.78–4.73 (m, 2H), 4.13 (q, *J* = 7,1 Hz, 2H), 3.45 (d, *J* = 5,7 Hz, 2H), 2.36–2.32 (m, 2H), 2.06 (s, 4H), 1.74–1.55 (m, 2H), 1.52–1.43 (m, 27H), 1.27 (t, *J* = 7,1 Hz, 2H).

^13^C-NMR (300 MHz, CDCl_3_): δ [ppm] = 189.09, 172.22, 157.27, 124.41, 125.10, 77.35, 77.03, 76.71, 70.07, 53.20, 44.39, 31.59, 28.00, 21.93, 21.07, 14.20.

MS (ESI^+^): 612,4 [M + H]^+^, calculated for C_30_H_49_N_3_O_10_: 611,34 [M].

2-[3-(2-(2-ethoxy-3,4-dioxo-cyclobut-1-en-1yl)amino-1-carboxy-pentyl)-ureido]-pentanedioic acid (**13**)

Compound **12** (50 mg, 0.082 mmol) was stirred with a mixture of dichloromethane and trifluoracetic acid (2 mL, 1:1) at room temperature for 2 hours. The solvent was evaporated under reduced pressure. The product was obtained as a colorless oil (30.2 mg, 0.068 mmol, 83%) and used without further purification.

^1^H-NMR (300 MHz, D_2_O): δ [ppm] = 4.75–4.65 (m, 2H), 4.30–4.12 (m, 2H), 3.59 (dt, *J* = 23.5 Hz, 6.6 Hz, 1H), 3.48 (t, *J* = 6.6 Hz, 1H) 2.49 (t, *J* = 7.3 Hz, 2H), 2.16 (dtd, *J* = 15.3 Hz, 7.4 Hz, 5.2 Hz, 1H), 2.04–1.90 (m, 1H) 1.86–1.75 (m, 2H), 1.73–1.46 (m, 3H), 1.41 (dt, *J* = 7.1 Hz, 3.6 Hz, 5H).

^13^C-NMR (300 MHz, D_2_O): δ [ppm] = 188.86, 182.94, 177.13, 176.95, 176.05, 173.15, 159.08, 70.41, 52.91, 52.48, 30.26, 29.91, 28.86, 26.15, 21.59, 14.95.

MS (ESI^+^): 444,2 [M + H]^+^, calculated for C_18_H_25_N_3_O_10_: 443,15 [M]^+^.

DATA^5m^.SA.KuE (**14**)

Compound **9** (30 mg, 0.067 mmol) and compound **13** (42 mg, 0.095 mmol) were dissolved in 0.5 M phosphate buffer (pH 9, 1 mL). The pH was adjusted to **9** and stirred for two days at room temperature. The crude product was purified by HPLC (column: LiChrospher 100 RP18 EC (250 × 10 mm) 5 μ, flow rate: 5 mL/min, H_2_O/MeCN + 0.1% TFA, 9 to 15% MeCN in 20 min, Rt = 10.1 min) to obtain DATA^5m^.SA.KuE as a white powder (5.26 mg, 0.0062 mmol, 10%).

MS (ESI^+^): 843.3 [M + H]^+^, 422.2 [M + 2H]^2+^, 441.2 [M + K + H]^2+^, calculated for C_35_H_54_N_8_O_16_: 842.37 [M]^+^.

### 4.3. Radiolabeling

For radiochemical evaluation, gallium-68 was eluted from a ^68^Ge/^68^Ga-generator (ITG Graching, Munich, Germany) and purified manually with ethanol-based post-processing to separate iron, zinc, and germanium impurities [44].

Radiolabeling was performed in 0.4 mL 1 M ammonium acetate buffer at pH 5.5. Reactions were carried out with different amounts of precursor (5, 10, 15, 60 nmol) and at different temperatures (RT, 50 °C, and 70 °C) with 30–50 MBq gallium-68. The pH was controlled at the start and after the labeling. For reaction control, radio-TLC (TLC Silica gel 60 F_254_ Merck) and citrate buffer pH 4 as mobile phase and radio-HPLC using an analytical HPLC 7000 series Hitachi LaChrom (Column: Merck Chromolith^®^ RP-18e, linear gradient of 5–95% MeCN (+0.1% TFA)/95–5% Water (+0.1% TFA) in 10 min). TLCs were measured in a TLC imager CR-35 Bio Test-Imager (Elysia-Raytest, Angleur, Belgium) with the analysis software AIDA (Elysia-Raytest, Angleur, Belgium).

Radiolabeling of AAZTA^5^.SA.KuE with scandium-44 and lutetium-177 was performed according to the literature [17].

### 4.4. In Vitro Stability Studies

Complex stability studies were performed in human serum (HS, human male AB plasma, USA origin, Sigma Aldrich) and phosphate buffered saline (Sigma Aldrich). First, 8–10 MBq of the labeled compound were added to 0.5 mL of the media. Afterwards, 30, 60, and 120 min aliquots were taken to evaluate the radiochemical stability. The percentage of complexed gallium-68, which corresponds to the percentage of *in vitro* radiochemical stability, was determined via radio-TLC. The studies were carried out in triplicate.

### 4.5. In Vitro Binding Affinity

PSMA binding affinity was determined according to the literature [39]. LNCaP-cells (purchased from Sigma-Aldrich) were cultured in RPMI 1640 (Thermo Fisher Scientific) supplemented with 10% fetal bovine serum (Thermo Fisher Scientific), 100 μg/ml streptomycin, and 100 units/mL penicillin at 37 °C in 5% CO_2_ in a humidified atmosphere. The medium was changed approximately every 3 days. Cells in exponential phase of growth were harvested by a 5 min treatment with a 0.05% trypsin–0.02% EDTA solution and neutralized with serum-containing medium prior to counting.

10^5^ LNCaP cells per well were applied in MultiScreen_HTS_ DV Filter Plates (Merck Millipore) and incubated with 0.75 nM [^68^Ga]Ga-PSMA-10 in the presence of 12 increasing concentrations of the non-labeled SA-conjugated compounds. After incubation at room temperature for 45 min, cells bound on the filter plates were washed several times with ice-cold PBS to remove free radioactivity. The cell-bound activity was determined by punching out the filters and transferring them into individual tubes for measurement in a γ-counter (2480 WIZARD^2^ Automatic Gamma Counter, PerkinElmer, Waltham, MA, USA). Data were analyzed in GraphPad Prism 9 using nonlinear regression. Experiments were replicated 4-times.

### 4.6. Internalization Ratio

Internalization ratio was determined according to the literature [45,46]. Prior to seeding cells, 24-well plates were coated with 0.1% poly-l-lysine (Sigma-Aldrich) in PBS for 20 min at room temperature. Subsequently, 10^5^ LNCaP cells in 1 mL RPMI 1640 Medium were added in each well and incubated for 24 h at 37 °C. Then, 250 µL of the ^68^Ga-labeled compounds in Opti-MEM™ I Reduced Serum (ThermoFisher) were added to each well to a final concentration of 30 nM. The plates were then incubated for 45 min at 4 °C and 37 °C respectively either with or without adding PMPA (Sigma-Aldrich) to a final concentration of 500 µM. The supernatant was removed and the cells were washed several times with ice-cold PBS. Afterwards, cells were incubated twice with 50 mM glycine buffer pH 2.8 for 5 min to remove the surface-bound radioactivity. In order to determine the internalized fraction of the compounds, cells were lysed by incubation with 0.3 M NaOH for 10 min.

### 4.7. Animal Studies

Six- to eight-week-old male BALB/cAnNRj (Janvier Labs) were inoculated subcutaneously with 5 × 10^6^ LNCaP cells in 200 µL 1:1 (*v*/*v*) Matrigel/PBS (Corning^®^). *In vivo* and *ex vivo* experiments are conducted after tumors reached a volume of approximately 100 mm^3^.

LNCaP-xenografts were anesthetized with 2% isoflurane prior to i.v. injection of 0.5 nmol of the radiolabeled compounds. The specific activities of the tracers were approximately 10 MBq/nmol, 6 MBq/nmol, and 15 MBq/nmol of gallium-68-labeled compounds, [^44^Sc]Sc-AAZTA^5^.SA.KuE and [^177^Lu]Lu-AAZTA^5^.SA.KuE, respectively. For blocking experiments, mice were co-injected with 1.5 µmol PMPA/mouse.

Biodistribution studies. The number of animals used in this study was: [^68^Ga]Ga-DATA^5m^.SA.KuE n = 5; [^44^Sc]Sc-AAZTA^5^.SA.KuE n = 2; [^177^Lu]Lu-AAZTA^5^.SA.KuE n = 2; [^68^Ga]Ga-PSMA-11 n = 2; [^44^Sc]Sc-AAZTA^5^.SA.KuE + PMPA n = 1. Animals were sacrificed 1 h p.i. Organs of interest were collected and weighed. The radioactivity was measured and calculated as a decay-corrected percentage of the injected dose per gram of tissue mass %ID/g.

MicroPET-imaging. After i.v. injection of the labeled compounds, anesthetized mice (one mouse for each group) were placed in the prone position in a nanoScan^®^ PET/MR (Mediso). MRI measurements were performed followed by a static PET scan with the nanoScan PET/MRI (Mediso, Budapest, Hungary). PET data were reconstructed with Teratomo 3D (four iterations, six subsets, voxel size 0.4 mm), co-registered to the MR, and analyzed with Pmod software (version 3.6) (PMOD Technologies LLC, Zürich, Switzerland) Material Map for co-registration of the PET scan; 3D Gradient Echo External Averaging (GRE-EXT), Multi Field of View (FOV); slice thickness, 0.6 mm; TE, 2 ms; TR, 15 ms; flip angle, 25 deg.

## 5. Conclusions

In summary, the synthesized hybrid chelator-based PSMAradiopharmaceuticals DATA^5m^.SA.KuE and AAZTA^5^.SA.KuE could be labeled at mild conditions with high radiochemical yields. The stability of the labeled compounds in PBS and human serum was demonstrated. Both SA.KuE conjugates displayed good PSMA binding affinities in LNCaP cells along with good internalization ratios. Additionally, [^68^Ga]Ga-DATA^5m^.SA.KuE, [^44^Sc]Sc-AAZTA^5^.SA.KuE, and [^177^Lu]Lu-AAZTA^5^.SA.KuE showed similar *in vivo* behavior, suggesting that the exchange of either the chelator or the nuclide does not impact the pharmacokinetic of the investigated compounds. This finding renders [^44^Sc]Sc-AAZTA^5^.SA.KuE and [^177^Lu]Lu-AAZTA^5^.SA.KuE an interesting pair for theranostic application. Tumor accumulation of the tested PSMA radioligands was similar to that of [^68^Ga]Ga-PSMA-11, although lower than the value reported in literature for PSMA-617. The decreased kidney uptake of the SA.KuE conjugates is noteworthy, which could be a major benefit in reducing irradiation of the kidneys, resulting in lower nephrotoxicity and improved tolerability.

## Figures and Tables

**Table 1:** IC_50_ values of the investigated compounds. Data represented as mean ± SD (n = 3).

Compound	IC_50_ [nM]
DATA^5m^.SA.KuE	51.1 ± 5.5
AAZTA^5^.SA.KuE	52.1 ± 4.0
PSMA-11	26.2 ± 2.4

## Data Availability

The data presented in this study are available on request from the corresponding author.

## Sample Availability

Not available.
